# Exploring the Association Between Age Groups and Success Patterns in Platelet-Rich Plasma Therapy: A Cohort Study

**DOI:** 10.7759/cureus.53418

**Published:** 2024-02-01

**Authors:** Shilpa Dutta, Akash More, Deepti Shrivastava, Namrata Choudhary, Mayur Wanjari, Vaibhav P Anjankar, Ashish Anjankar, Mehak Chopra, Shivani Khemani

**Affiliations:** 1 Clinical Embryology, Datta Meghe Institute of Higher Education and Research, Wardha, IND; 2 Obstetrics and Gynecology, Jawaharlal Nehru Medical College, Datta Meghe Institute of Higher Education and Research, Wardha, IND; 3 Research and Development, Jawaharlal Nehru Medical College, Datta Meghe Institute of Higher Education and Research, Wardha, IND; 4 Anatomy, Jawaharlal Nehru Medical College, Datta Meghe Institute of Higher Education and Research, Wardha, IND; 5 Biochemistry, Jawaharlal Nehru Medical College, Datta Meghe Institute of Higher Education and Research, Wardha, IND; 6 Public Health, Parul Institute of Public Health, Vadodara, IND

**Keywords:** clinical pregnancy, infertility, endometrium, hormone replacement therapy, platelet-rich plasma therapy

## Abstract

Objective

This study aimed to comprehensively examine the correlation between success trends in platelet-rich plasma (PRP) therapy and the advancing age of patients undergoing fertility interventions.

Methods

Female participants were categorized randomly into five age groups undergoing PRP or conventional hormone replacement therapy. Procedures included controlled ovarian stimulation, escalating estrogen dosage, gonadotrophin injections, and embryo transfer post-ovulation trigger. A pivotal PRP intervention was provided to half of the age sub-groups, and endometrial thickness was assessed 24 hours prior to embryo transfer. Statistical analysis employed SPSS 26.0 for Windows Student Version (IBM Inc., Armonk, New York), incorporating descriptive statistics, one-way analysis of variance (ANOVA), Tukey's honestly significant difference (HSD) test to explore age-PRP success relationships (p<0.05).

Results

The study, involving 60 participants, revealed a balanced patient distribution across age groups, with 20-30 age groups contributing 23.33% each. Baseline characteristics showed no significant differences between PRP and hormone replacement therapy (HRT) groups. Post-intervention, PRP demonstrated consistently higher endometrial thickness (p<0.001) and clinical pregnancy rates (63.33%) compared to HRT (40%). These findings suggest a positive association between PRP therapy and improved outcomes, particularly in younger age cohorts.

Conclusion

The study challenges traditional perspectives on hormonal influences in fertility, highlighting a potential link between PRP therapy and favorable outcomes among younger age groups. Improved endometrial thickness and clinical pregnancy rates in the PRP group emphasize the need for further exploration of PRP's mechanisms and applications in reproductive medicine.

## Introduction

The natural process that we're bestowed upon by evolution has also been undergoing various integral changes following the advancement in technology to improve our lifestyle all around the world. The changes have been quite detrimental to the innate processes per se [1]. One of the current detrimental issues that we are facing is infertility. It has been estimated that more than 180 million families have been suffering from infertility across the globe [1,2]. Researchers have discovered multiple reasons contributing to infertility. Out of these, the one that is currently gaining quite an insight is endometrium. It has been globally accepted that positive clinical pregnancy outcome establishment lies in the thickness of the endometrial layer to sustain a healthy conceptus [3]. Some researchers have pointed out that in order to have a hassle-free implantation in the endometrial layer, the lining should have a minimum width of 7mm [4]. If the lining is below the desired width, this generally results in miscarriages or recurrent implantation failure [4]. In the year 2015, breakthrough research was conducted on a couple who gave live birth to their baby through the use of platelet-rich plasma therapy [5]. Platelet-rich plasma therapy, also abbreviated as PRP, is the extraction of amplified platelets through differential centrifugation and is used upon the endometrium to enhance its thickness. The proposition behind using PRP therapy is that it is reportedly found to promote cellular proliferation, thus leading to enhanced growth of the endometrial lining [6]. 

PRP, also promulgated as autologous conditioned plasma, is an extract of platelet-rich protein procured from the whole, unprocessed blood from the human body. Platelets have reportedly been found to obtain several growth factors and cytokines, which stimulate healing properties in cells, thus promoting healthy cellular complexes. The concept originated in the hematological department in the 1970s [7]. It was first utilized as a transfusion commodity to treat thrombocytopenic patients. Subsequently, due to its wound-healing properties, it has been extensively used in sports for treating musculoskeletal injuries. Wounds have an affinity towards inflammatory biochemical habitat that diminishes the scope of healing in chronic ulcers. Further, it is also reported to have a higher proteolytic function, characterized by a reduction in the congregation of growth factor. Platelet-rich plasma has been employed as a fascinating treatment for perverse wounds. This is so because of the plethora of growth factors in PRP along with these angiogenic, mitogenic, and chemotactic effects [6,7]. 

Upon extensive utilization of platelet-rich plasma therapy in sports, it has gained the attention of the media, and subsequently, research was conducted in other medical areas to learn about its effect on them. Upon the live birth that occurred following the use of PRP in 2015, several kinds of research on the application of PRP for improvement in endometrium thickness were conducted on different apathies related to reproductive biology, and most have pointed out a positive result of having pregnancy outcomes following the use of it [3-6]. PRP is generally extracted following the withdrawal of peripheral blood from the patient and centrifuged differentially under two spins to collect the desired pallet of platelet for use. The normal physiological mark that is present in the blood for platelets is between 150,000 and 450,00 platelets per microliter of blood [7]. For the procedure requirement, the amount of amplified platelet needed is two to three times more than the normal level. The centrifugation helps to reach the desired level for infusion in the endometrium [8]. Once the processed pallet is infused into the endometrium, there have been significant reactions that increase the thickness of the endometrium, forming the ground for implantation. Though different experimental processes have been trialed by many researchers to date, owing to the sample size being restrictively studied in most research, the results have not been commercially validated, and the procedure is still in the experimental stage [9]. Hence, this study has been done to evaluate the correlation between the success rate in PRP therapy performed on patients along with the subsequent increase in the advancement of age based on a relatively large sampling size to make it available for commercial use.

## Materials and methods

Study design and population

This observational cohort study involved 60 eligible women with thin endometrium and a history of recurrent implantation failure, recruited from the Wardha Test Tube Baby Centre (WTTBC) at Acharya Vinoba Bhave Rural Hospital (AVBRH), Sawangi, from February 2023 to January 2024.

Selection criteria

Inclusion criteria comprised women below 45 years who had experienced in vitro fertilization (IVF) failure due to thin endometrium and were planning platelet-rich plasma therapy along with in vitro fertilization at WTTBC. Exclusion criteria included women above 45 years, those participating in other experimental interventions for recurrent implantation failure, and those with uterine polyps or endometriosis intending to pursue in vitro fertilization with the assistance of a gestational carrier.

Ethical consideration

Prior to initiating any procedures, a comprehensive process of obtaining written informed consent was diligently undertaken, ensuring that only those individuals willingly committed to participation were enrolled in the study. This study was ethically approved by the Institutional Ethical Committee of Datta Meghe Institute of Higher Education and Research in its meeting on 02/02/2023 with reference number DMIHER(DU)/IEC/2023/609.

Methodology

In this study, the female patients were categorized into five age groups: 20-25, 25-30, 30-35, 35-40, and 40-45 years. Within each group, patients were further divided into two subcategories: one undergoing platelet-rich plasma (PRP) therapy, and the other conventional hormone replacement therapy.

All the enrolled patients underwent controlled ovarian stimulation. Hormone replacement therapy followed, involving an escalating oral estrogen dosage from 6mg/day on the third menstrual cycle day, incrementally reaching 12 mg/day. Gonadotrophin injection commenced on day three of menses, along with transvaginal ultrasound. Follitropin alfa was administered for a five-day stimulation period. If more than two follicles exceeded 14mm, follicle-stimulating hormone (FSH) injection ceased. Ovulation was triggered with 1000IU of human chorionic gonadotropin (hCG) 34-36 hours before ovarian aspiration. Continuous administration of injection cetrorelix and follitropin alfa was carried out until three to four follicles reached a size greater than 17mm. Estradiol (E2) and serum concentrations were examined on the day of hCG administration. Oocytes were retrieved after 34 hours of hCG injection via follicular aspiration and were performed according to standard protocol. Embryo transfer occurred 120 hours post oocyte retrieval, with patients receiving daily intramuscular progesterone. Twenty-four hours before the embryo transfer procedure, a pivotal intervention occurred for half of the subcategory patients in each age group. A volume of 15-20 ml of peripheral blood was meticulously withdrawn into a syringe pre-filled with an anticoagulant. This blood sample underwent a dual-phase centrifugation process, with the first centrifugation executed at 1000 rpm for 10 minutes, followed by the collection of the supernatant. This was then subjected to a second centrifugation at double the rpm for an equivalent timeframe. The resulting pellet was then judiciously infused into the uterine cavity using a catheter with global acceptance (0.5-1ml). After administration of platelet-rich plasma into half of the subcategories across different age groups, the study evaluated endometrial thickness. This assessment, conducted 24 hours after the PRP intervention, employed transvaginal ultrasonography to measure the thickness at the thickest part of the longitudinal axis of the uterus. Embryo transfer, conducted on the 21st day of the menstrual cycle, was followed by pregnancy validation through β-hCG level assessments. A comparative analysis of the data acquired from both groups of patients followed, and the results were graphically represented to provide a comprehensive understanding of the outcomes.

Statistical analysis

Descriptive statistics were employed to summarize the data for each age group, encompassing mean, standard deviation, and range. The success rate of platelet-rich plasma (PRP) therapy was computed as the percentage of patients who exhibited a noteworthy improvement in pain levels and functional status following the treatment. A one-way analysis of variance (ANOVA) was utilized to compare the mean success rates across distinct age groups. Subsequently, Tukey's honestly significant difference (HSD) test was executed as a post-hoc analysis to discern any significant differences among specific age groups. Moreover, a multivariate regression analysis was conducted to explore the relationship between age and the success rate of PRP therapy while adjusting for other potential confounding variables such as sex, medical history, and baseline pain levels. All statistical analyses were carried out using SPSS 26.0 for Windows Student Version (IBM Inc., Armonk, New York), and statistical significance was established at p<0.05.

## Results

Table 1 details the distribution of patients across different age groups in a study investigating the correlation between the success trend in Platelet-Rich Plasma (PRP) therapy and patient age. The age categories, ranging from 20 to 45 years in five-year intervals, reveal a relatively balanced representation. Notably, the 26-30 and 31-35 age groups each contribute 23.33% (14/60) to the total patient count, with 14 individuals in each bracket. The 36-40 age group follows closely with a 20% (12/60) share, comprising 12 patients. Similarly, both the 20-25 and 41-45 age groups account for 16.67% (10/60) of the total, encompassing 10 patients each. This demographic breakdown, totaling 60 participants, establishes a foundational understanding of the study population, laying the groundwork for further analysis to explore potential correlations between patient age and the success of platelet-rich plasma therapy.

**Table 1:** Distribution of patients in each age group

Age group (years)	Frequency	Percent (%)
20-25	10	16.67 (10/60)
26-30	14	23.33 (14/60)
31-35	14	23.33 (14/60)
36-40	12	20 (12/60)
41-45	10	16.67 (10/60)
Total	60	100

The data from Table 2 reveals that FSH levels in the PRP group ranged from 1.56 to 9.73 mIU/L, with a mean of 5.645 and a standard deviation of 2.62062864. In comparison, the hormone replacement therapy (HRT) group exhibited FSH levels between 1.9 and 7.65 mIU/L, with a mean of 4.775 and a standard deviation of 2.91664905. Notably, the difference in FSH levels between the two groups did not reach statistical significance (p=0.228). Similarly, for Luteinizing hormone (LH) levels, anti-Mullerian hormone (AMH) levels, and their respective statistical parameters, no significant differences were observed between the PRP and HRT groups.

**Table 2:** Distribution of baseline characteristics p-value<0.05 is considered to be significant PRP - platelet-rich plasma; HRT - hormone replacement therapy; FSH - follicle-stimulating Hormone; LH - Luteinizing hormone; AMH - anti-Mullerian hormone; mIU/L - milli-international units per liter; IU/L - international units per liter; g/dl - nanograms per deciliter

Variables	PRP group (N=30)				HRT group (N=30)				p-value
	Minimum	Maximum	Mean	Std. deviation	Minimum	Maximum	Mean	Std. deviation	
LH level (IU/L)	9.4	55.4	32.4	19.0855	11.91	54.21	33.06	17.2689	0.889
FSH level (mIU/L)	1.56	9.73	5.645	2.620629	1.9	7.65	4.775	2.916649	0.228
AMH level (ng/dl)	1.1	3.7	2.4	1.061446	1	3.67	2.335	1.07632	0.815

In examining the relationship between the success trend in platelet-rich plasma (PRP) therapy and the advancement in age among patients, Table 3 illustrates the distribution of key characteristics across different age groups. The variables include body mass index (BMI), duration of infertility, and antral follicle count (AFC) for both the PRP and hormone replacement therapy (HRT) groups. Noteworthy patterns emerge as one evaluates these parameters across age categories. For instance, in the 20-25 age group, both the PRP and HRT groups exhibit similar BMI and AFC values, while the duration of infertility is marginally higher in the HRT group. Conversely, in the 31-35 age group, the PRP group demonstrates a slightly higher BMI and AFC compared to the HRT group, with a marginal difference in the duration of infertility.

**Table 3:** Patients' characteristics according to different age groups BMI - body mass index; AFC - antral follicular count; PRP - platelet-rich plasma; HRT - hormone replacement therapy

Age group (years)	BMI (kg/m^2^)		Duration of infertility (years)		AFC (n)	
	PRP	HRT	PRP	HRT	PRP	HRT
20-25	24.2±3.64	24.2±3.49	7.35±4.31	7.75±4.02	12.05±7.05	13.21±7.29
26-30	25.6±4.65	24.8±3.85	6.72±4.62	6.81±4.29	12.05±7.06	12.05±7.06
31-35	24.8±3.86	25.3±4.69	8.05±4.63	7.95±4.14	12.05±7.07	10.95±6.63
36-40	26.4±4.17	26.2±4.67	7.84±4.64	8.11±4.24	11.15±6.44	12.2±7.67
41-45	24.9±3.81	25.7±3.12	7.38±4.65	6.92±4.23	12.81±7.29	12.15±7.25

**Figure 1:**
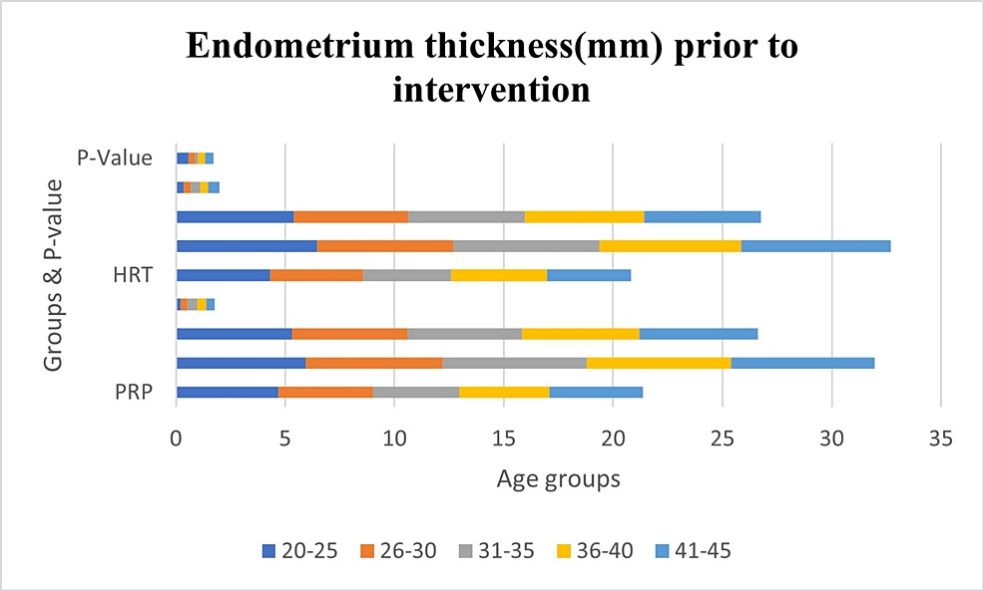
Graphical representation Bar graph representing endometrium thickness (mm) prior to intervention. p-value<0.05 is considered to be significant. HRT- hormone replacement therapy; PRP - platelet-rich plasma therapy

The provided data in Table 4 outlines the endometrium thickness (mm) prior to intervention across different age groups in the platelet-rich plasma (PRP) and hormone replacement therapy (HRT) groups as part of a study on the correlation of the success trend in platelet-rich plasma therapy with the advancement in the age of patients. Figure 1 is the representation of the data in the form of a bar graph. In examining the mean and standard deviation values, it is observed that, in general, there are minimal variations in endometrium thickness between the PRP and HRT groups within each age category. Notably, no statistically significant differences are observed when comparing the mean endometrium thickness values between the two treatment groups in the various age groups, as indicated by the p-values ranging from 0.125 to 0.567. These findings suggest that, at least in terms of endometrium thickness prior to intervention, there is no substantial divergence between the PRP and HRT groups across different age brackets.

**Table 4:** Distribution of endometrial thickness (mm) prior to intervention p-value<0.05 is considered to be significant PRP - platelet rich plasma; HRT - hormone replacement therapy

Age Groups(Years)	PRP				HRT				p-value
	Minimum	Maximum	Mean	Std. Deviation	Minimum	Maximum	Mean	Std. Deviation	
20-25	4.69	5.95	5.32	0.21	4.31	6.47	5.39	0.36	0.567
26-30	4.33	6.25	5.29	0.32	4.24	6.22	5.23	0.33	0.32
31-35	3.94	6.61	5.23	0.46	4.04	6.68	5.36	0.44	0.125
36-40	4.13	6.59	5.36	0.41	4.38	6.48	5.43	0.35	0.321
41-45	4.27	6.55	5.41	0.38	3.84	6.84	5.34	0.5	0.397

**Figure 2:**
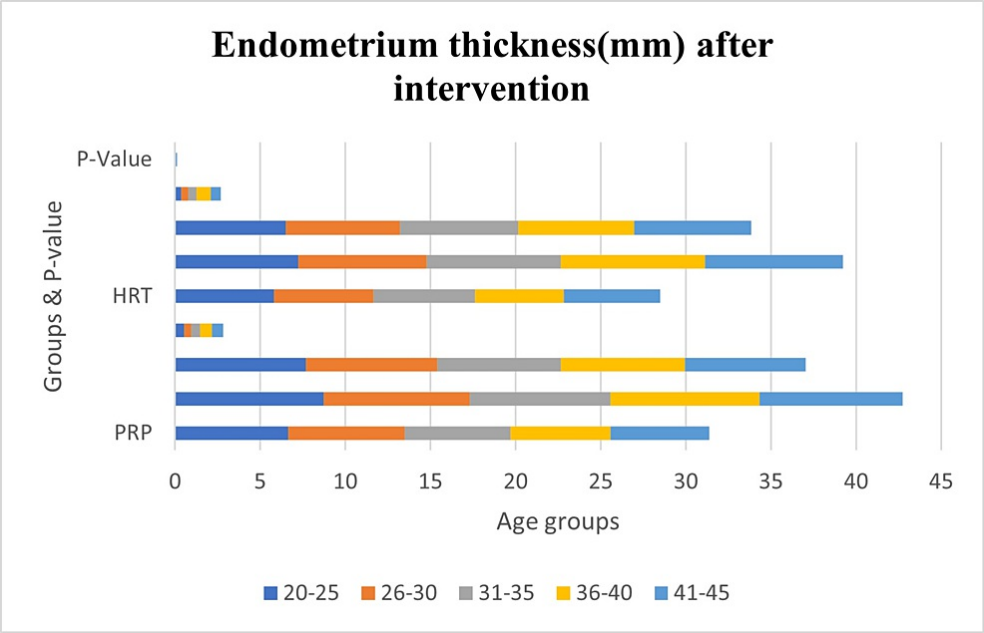
Graphical representation Bar graph representing endometrium thickness (mm) after intervention. p-value <0.05 is considered to be significant. HRT - hormone replacement therapy; PRP - platelet-rich plasma therapy

Table 5 denotes data regarding endometrium thickness (mm) after intervention in the platelet-rich plasma (PRP) and hormone replacement therapy (HRT) groups across various age groups, presents compelling insights for the study on the correlation of the success trend in platelet-rich plasma therapy with the advancement in the age of patients. Figure 2 is the representation of the data in the form of a bar graph. The mean endometrium thickness values post-intervention exhibit notable differences between the PRP and HRT groups within each age category. Specifically, in the 20-25 and 26-30 age groups, the PRP group demonstrates significantly higher mean endometrium thickness compared to the HRT group, as indicated by p-values of <0.001. In the 31-35 age group, although the difference is statistically significant (p=0.02), the PRP group still maintains a higher mean endometrium thickness after intervention. Similarly, in the 36-40 age group, the PRP group shows a statistically significant advantage over the HRT group (p=0.03). While the 41-45 age group does not reach statistical significance (p=0.06), there is a trend suggesting a potential benefit in favor of the PRP group among younger age groups.

**Table 5:** Distribution of endometrial thickness (mm) after intervention p-value <0.05 is considered to be significant PRP - platelet-rich plasma therapy; HRT - hormone replacement therapy

Age Groups(Years)	PRP				HRT				p-value
	Minimum	Maximum	Mean	Std. Deviation	Minimum	Maximum	Mean	Std. Deviation	
20-25	6.64	8.72	7.68	0.52	5.8	7.24	6.52	0.36	<0.001
26-30	6.86	8.58	7.72	0.43	5.86	7.54	6.7	0.42	<0.001
31-35	6.22	8.26	7.24	0.51	5.96	7.88	6.92	0.48	0.02
36-40	5.87	8.75	7.31	0.72	5.2	8.48	6.84	0.82	0.03
41-45	5.77	8.41	7.09	0.66	5.67	8.07	6.87	0.6	0.06

Table 6 denotes the clinical pregnancy outcomes, determined by β-hCG tests, revealing varying rates between the platelet-rich plasma (PRP) and hormone replacement therapy (HRT) groups across age categories. In the 20-25, 26-30, 31-35, and 36-40 age groups, PRP consistently shows higher clinical pregnancy rates than HRT. The 41-45 age group also indicates a higher rate in the PRP group. Percentage data further supports this trend, with a 63.33% (19/30) clinical pregnancy rate in PRP versus 40% (12/30) in HRT. These findings suggest a positive correlation between PRP therapy and improved clinical pregnancy outcomes, emphasizing the relevance of patient age in assessing success trends. Examining endometrial characteristics post-intervention, PRP consistently demonstrates a higher mean endometrial thickness across age groups compared to HRT. Significant differences are noted in the 20-25, 26-30, and 31-35 age groups, with PRP maintaining superiority. Although not statistically significant in the 41-45 age group, a potential trend favors PRP intervention, particularly in younger age cohorts. These results imply a positive association between PRP therapy and enhanced endometrial development, which is crucial for successful implantation and pregnancy.

**Table 6:** Distribution of patients according to human chorionic gonadotropin (β-hCG) report PRP - platelet-rich plasma; HRT - hormone replacement therapy; hCG - human chorionic gonadotropin

Age groups (years)	Clinical pregnancy (+ve β-hCG test)	
	PRP	HRT
20-25	3	2
26-30	5	4
31-35	5	3
36-40	4	2
41-45	2	1
Per cent	63.33% (19/30)	40% (12/30)

## Discussion

The scientific discussion surrounding the correlation between the success rate of plasma-rich platelet (PRP) therapy and the advancing age of patients in reproductive medicine is rooted in a rich historical context. Originating in the 1970s as a treatment for thrombocytopenic patients, PRP therapy evolved into a promising intervention for musculoskeletal injuries in sports and subsequently gained traction in reproductive biology [1]. The literature review establishes a solid foundation, referencing pivotal studies such as crucial studies, including Chang's work on thin endometrial linings, demonstrating a 100% success rate in achieving pregnancy and live births post-PRP administration [5]. Eftekar et al.'s randomized controlled trials with 66 individuals showed significantly greater endometrial thickness in the PRP group, leading to higher success in frozen embryo transfer (FET) compared to hormone replacement therapy (HRT) [10]. Retrospective analyses by Coksuer et al. on 70 women with a failure of multiple implantation failures due to idiopathic reasons found that there has been a significant increase in lining thickness along with increased pregnancy rates with live births in patients who underwent PRP compared to the latter group [11].

Frantz et al.'s retrospective study on 24 IVF cycle patients demonstrated positive pregnancy outcomes with PRP, irrespective of the initial endometrial thickness [12]. Kusumi et al.'s self-controlled trial on Japanese patients with thin endometrium favored the use of PRP based on standard deviation calculations [13]. Hajipour et al.'s recent study emphasized the need for proper guidelines in PRP administration to commercialize it with minimal side effects [14]. Molina et al.'s 2018 study on 19 patients with refractory thin endometrium reported significant endometrial growth (>9 mm) with a 73.7% pregnancy rate and a 26.3% live birth rate [15]. Zamaniyan et al.'s trial on 98 patients with recurrent implantation failure showed a significant increase in implantation rate, clinical pregnancy, and live birth following PRP infusion [16].

Collectively, these studies emphasize the positive impact of PRP therapy on endometrial thickness and clinical pregnancy outcomes. Our study adds nuanced insights by examining hormonal profiles, BMI, duration of infertility, antral follicle count (AFC), and endometrial characteristics across age groups. The consistently higher mean endometrial thickness in the PRP group, especially in younger cohorts, suggests a potential positive association between PRP therapy and enhanced endometrial development crucial for successful implantation and pregnancy. While acknowledging the limitations of retrospective studies, our findings contribute to the discourse on personalized treatment approaches in reproductive medicine. Prospective, randomized controlled trials are imperative to address ethical considerations, potential side effects, and standardization of PRP therapy procedures. In conclusion, our study provides valuable insights into the potential of PRP therapy in addressing infertility, highlighting the need for ongoing research and validation.

Strengths and limitations

This cohort study represents the first conducted study, as per available literature, that has attempted to assess the most applicable age group in which PRP shows the maximum results in patients suffering from recurrent implantation failure due to a thin endometrium. The balanced distribution of patients across age groups helps ensure that the results are not skewed by an overrepresentation of a particular age category, enhancing the generalizability of the findings. There has been a significant correlation between the success of PRP treatment and pregnancy outcomes, especially in the younger age cohort.

The first notable limitation of this study is that it is based on a significantly small sample size. This may limit the generalization of the results of this study to a larger general population. Also, this study is based on a single-center analysis, and hence, it may introduce institutional biases. Therefore, further research should be conducted with larger sample sizes and multiple-center analyses to strengthen the results of this study.

## Conclusions

In summary, our study investigating the correlation between platelet-rich plasma (PRP) therapy success and patient age reveals a nuanced landscape in fertility interventions. Contrary to expectations, hormonal profiles in both PRP and hormone replacement therapy (HRT) groups exhibit comparable levels across age brackets, suggesting that hormonal factors may not be the primary determinants of PRP therapy success. Subtle variations in BMI, duration of infertility, and antral follicle count (AFC) within age groups emphasize the intricate interplay of factors influencing treatment outcomes. Notably, our analysis uncovered a significant association between PRP therapy and improved endometrial development. The PRP group consistently demonstrates higher mean endometrial thickness across age categories, with statistically significant differences in younger age cohorts. These findings imply a positive link between PRP intervention and enhanced endometrial conditions critical for successful implantation and pregnancy. Clinical pregnancy outcomes echo these trends, with the PRP group consistently exhibiting higher rates across all age groups. The notable difference in clinical pregnancy rates underscores the potential correlation between platelet-rich plasma therapy and favorable fertility outcomes. Overall, our study contributes valuable insights to the understanding of PRP therapy in reproductive medicine, emphasizing the need for further exploration of its mechanisms and applications in enhancing fertility.
